# Is an inotrope score a predictor of mortality and morbidity in children with septic shock?

**DOI:** 10.1186/cc14227

**Published:** 2015-03-16

**Authors:** E Sevketoglu, A Anil, S Kazanci, O Yesilbas, M Akyol, S Bayraktar, N Aksu, S Hatipoglu, M Karabocuoglu

**Affiliations:** 1 Bakirkoy Dr. Sadi Konuk Research and Training Hospital, Bakýrkoy, Turkey; 2 Izmir Katip Celebi University Medicine Faculty, Izmir, Turkey; 3 Haseki Research and Training Hospital, Istanbul, Turkey; 4 Tepecik Research and Training Hospital, Izmir, Turkey; 5 Sisli Memorial Hospital, Istanbul, Turkey

## Introduction

Inotropes and vasoactive drugs in septic shock are commonly used to maintain cardiac output, tissue perfusion and oxygenation. We undertook this study with the purpose of evaluating an inotropic score as a predictor of mortality and morbidity among children who diagnosed septic shock.

## Methods

A multicenter retrospective chart review was performed in two pediatric ICUs. A total of 93 children with septic shock were recruited. Hourly doses of following inotropes were recorded for the first 48 hours after admission: dopamine, dobutamine, adrenaline and noradrenaline. The inotrope score for every hour, minimum, maximum and mean values for the first 24 hours, and subsequent 24 hours were calculated. In our analysis, the inotrope score was calculated as described by Wernovsky. We expanded this formula to include norephinephrine as follows: Wernovsky Inotrope Score = dopamine dose (μg/kg/minute) + dobutamine dose (μg/kg/minute) + 100 × epinephrine dose (μg/kg/ minute). Our adjusted inotrope score = Wernovsky Inotrope Score + 100 × norepinephrine dose (μg/kg/minute).

## Results

Forty-two of 93 patients died. Ages and sex were not different between survivors and nonsurvivors. Significantly higher mean and maximum inotropic score for the first 24 hours and first 48 hours were found in nonsurvivors than those of survivors (*P *< 0.05). Using 15 as a cutoff point for predicting mortality, the sensitivity and specificity were 69.76% and 50.98% respectively. The association between Prism scores and minimum, mean and maximum inotrope scores were statistically significant for 0 to 24 hours, 25 to 48 hours and 0 to 48 h. Mean 0 to 24 hours and maximum 0 to 48 hours inotrope scores were weakly associated with prolonged ICU stay (*P *= 0.047, *P *= 0.042 respectively). There were no significant relationships between inotrope scores and receiving mechanical ventilation.

## Conclusion

The mean and maximum inotropic scores in the first 24 hours and 0 to 48 hours are an independent predictor of mortality in critically ill children with septic shock.

